# Role of melatonin in prevention of age-related hearing loss

**DOI:** 10.1371/journal.pone.0228943

**Published:** 2020-02-10

**Authors:** Lucieny Silva Martins Serra, Juliana Gusmão de Araújo, Ana Luiza Sarkis Vieira, Eduardo Magalhães da Silva, Rafael Rocha de Andrade, Selma Aparecida Souza Kückelhaus, André Luiz Lopes Sampaio

**Affiliations:** 1 Laboratory of Otorhinolaryngology Research, University of Brasilia, Brasília, Distrito Federal, Brazil; 2 Department of Tropical Medicine, University of Brasilia, Brasília, Distrito Federal, Brazil; 3 Faculty of Ceilândia, University of Brasilia, Brasília, Distrito Federal, Brazil; 4 Laboratory of Experimental Surgery, University of Brasilia, Brasília, Distrito Federal, Brazil; 5 Department of Morphology, University of Brasilia, Brasília, Distrito Federal, Brazil; Universidad de Chile, CHILE

## Abstract

**Introduction:**

Age-related hearing loss (ARHL) is a consequence of aging of the auditory system. The best known mechanism of cell death in ARHL is apoptosis due to increased production of reactive oxygen species. In this context, it is hypothesized that melatonin, owing to its high antioxidant potential and its action in the mitochondria, helps prevent or delay outer hair cell dysfunction (HCD).

**Aims:**

To evaluate the effect of melatonin on the prevention of HCD dysfunction in the ARHL process in a susceptible murine C57BL/6J model.

**Method:**

C57BL/6J animals were divided into two groups: control (CG) and melatonin (MG). The CG received a saline and ethanol solution and the MG, melatonin (10 mg/kg/day). The solutions were offered daily (50 μl) orally over a 10-month period. Distortion Product Otoacoustic Emissions (DPOAE) measurements were conducted once a month.

**Results:**

There was a decrease in DPOAE values in both groups over time and a differentiation between them from the 10th month of life onwards. At 10 months, the MG maintained higher DPOAE values than the CG at all frequencies tested.

**Conclusion:**

The use of melatonin has otoprotective effects on HCD in the ARHL process in the C57BL/6J model.

## Introduction

Age-related hearing loss (ARHL) is an irreversible neurosensory disorder characterized by decreased hearing threshold sensitivity and speech comprehension skills. This condition generally arises during the fourth decade of life with progressive worsening with age [1]. It affects approximately 25–40% of individuals over the age of 65 and 80% of those over 85 [2,3]. Some studies have shown an association between ARHL and depression, gait difficulties and cognitive decline [4–6].

ARHL is a multifactorial (environmental and genetic) condition that involves both cochlear hair cells and neurons and can cause cell degeneration in the stria vascularis [4,6–8]. Throughout life, the most common and irreversible aging effect on the auditory system is the death of cochlear hair cells, which usually occurs by apoptosis in response to oxidative stress [2,9–11]. A recurrent complaint of senile individuals is difficulty with speech comprehension, especially in noisy environments [12].

Antioxidant substances have potential for the prevention or delay of hair cell death and have been the subject of recent studies on deafness and aging [7,12–14]. Among these substances, melatonin (N-acetyl-5-methoxytryptamine) has shown therapeutic promise. Melatonin originates from serotonin, and is the primary hormone of the pineal gland, where it is synthesized by pinealocytes with tryptophan as its main precursor. Melatonin is also synthesized locally in extrapineal tissues and organs [15,16]. It stimulates the production of various enzymes that protect cells, lipids, proteins and DNA from oxidative damage and crosses many morphophysiological barriers, increasing its cross-tissue effectiveness in neutralizing reactive oxygen species [15,17]. Due to these antioxidant characteristics, melatonin has already shown promise in the field of aging, where numerous studies have investigated the role of melatonin on different cellular mechanisms associated with aging [18,19].

Melatonin has been shown to be effective in the prophylaxis of cochlear damage caused by noise [20] as well as in improving the ototoxicity caused by cisplatin [21]. As age increases, melatonin production decreases, depriving the body of one of its most potent antioxidant compounds, which may favor the emergence of ARHL.

Regarding hearing loss, *in vivo* studies are important for evaluating the effect of antioxidant substances on the auditory system. The C57BL/6J mouse presents a classic form of early hearing loss that is similar to that in humans in the aging process (presbycusis). In addition, this strain develops other conditions commonly associated with aging such as diet-induced obesity, atherosclerosis, type-2 diabetes and low bone density [22,23].

The auditory function evaluation of these animals can be performed using distortion product otoacoustic emissions (DPOAE), which are acoustic signals recorded in the external auditory canal and generated by the cochlea in response to a stimulus. These responses have been verified over a range of frequencies and have been shown to change during the process of aging, thus being a reliable method for ARHL-related studies [24].

Since presbycusis is one of the main concerns of the aging population which leads to functional impairment, this study aimed to evaluate the effect of melatonin on the prevention of cochlear cell dysfunction in elderly C57BL/6J mice.

## Material and methods

### Type of study and animals

This was an experimental, prospective and interventional study of 32 male C57BL/6J mice acquired from the vivarium at the University of Campinas (UNICAMP).

The animals were obtained when they were four weeks old and were housed in the vivarium of the University of Brasilia for another four weeks of adaptation. During this time, the mean room temperature was 25±3°C, natural sleep and wake cycles (12:12) were induced, and mice were housed in environmentally enriched cages with access to water and balanced feed *ad libitum*.

After the adaptation period, animals were kept at the same vivarium and were handled according to the rules of the Brazilian College of Animal Experimentation -COBEA, 1990 [25].

After completion of data collection at 15 months of age, the animals were sacrificed by placing them in a hermetically sealed box containing a transparent display with a CO_2_ concentration maintained at 40%. Death of the animal was confirmed by the absence of corneal reflex and heartbeat [25].

### Study and treatment groups

The animals were evaluated using otoscopy. Animals presenting signs of otitis externa, acute otitis media or a meatus too narrow to adequately accommodate the probe of the otoacoustic emission equipment were removed from the study. The animals were then submitted to auditory screening using a distortion product otoacoustic emissions (DPOAE) apparatus (ERO-SCAN^®^—MAICO Diagnostics) in an acoustically isolated cabin under anesthesia with 65 mg/kg ketamine hydrochloride and 6.5 mg/kg xylazine. The animals showing the presence of DPOAEs in at least one ear were used in the experiment. The animals were divided into two groups:

- Control group (CG, n = 16), receiving 50 μl/day of saline and ethanol (12%), orally
- Melatonin group (MG, n = 16), receiving 10 mg/kg/day of melatonin (Sigma Aldrich^®^, St Louis, United States) diluted in 12% of ethanol, orally

Daily, each animal received by oral gavage a total volume of 50 μl administered through an automatic pipette. Both groups were supervised daily during the 10-month study period.

### Experimental procedure

Otoacoustic emissions were measured at the beginning of treatment and each month at 6, 8, 10 and 12 kHz. The function of outer cochlear hair cells was determined by DPOAE using an ERO-SCAN (MAICO Diagnostics). For the test, the animals were anesthetized with ketamine hydrochloride and xylazine, as described above. Before the recording of evoked otoacoustic emissions (EOAE), the animals were submitted to manual otoscopy for the evaluation of the auditory meatus and the tympanic membrane, and those presenting signs of otitis or earwax of difficult removal were excluded from the test. The DPOAE test was performed before treatment and immediately before sacrifice, using the 2f1-f2 frequency with an f1/f2 ratio of 1.22, presented at an average intensity of 65dB SPL for f1 and 55dB SPL for f2. The emission values obtained in the CG were considered as a reference for this strain. Acoustic signals registered on the external acoustic meatus were cochlear responses that aged mice are especially sensitive to. Therefore, DPOAE measurement is a reliable method for ARHL-related studies [24], since the acoustic signals recorded in the external auditory canal are considered cochlear responses to frequencies that are altered by aging.

### Statistical analysis

The normality of the distribution of variables was analyzed by the Kolmogorov-Smirnov test, while the variability of variances was analyzed by Bartlett’s test. ANOVA followed by the Student-Newman-Keuls or Kruskal-Wallis followed by the Dunn test were used to compare multiple parametric or non-parametric samples, respectively. Student’s t-test or the Mann-Whitney test were used to compare two independent normal or non-normal samples, respectively. Linear regression was used to evaluate the monthly DPOAE behavior.

The Prism^®^ Software Package program (GraphPad, USA, 2005) was used for performing the statistical tests and for graphical representations. Values of p<0.05 were considered statistically significant.

### Ethics statement

All procedures performed in studies involving animals were in accordance with the ethical standards of the institution or practice at which the studies were conducted. Ethical clearance and approval was granted by the University of Brasília Animal Ethics Committee with approval number 57/2017 (June 5, 2017).

## Results

**Fig 1:**
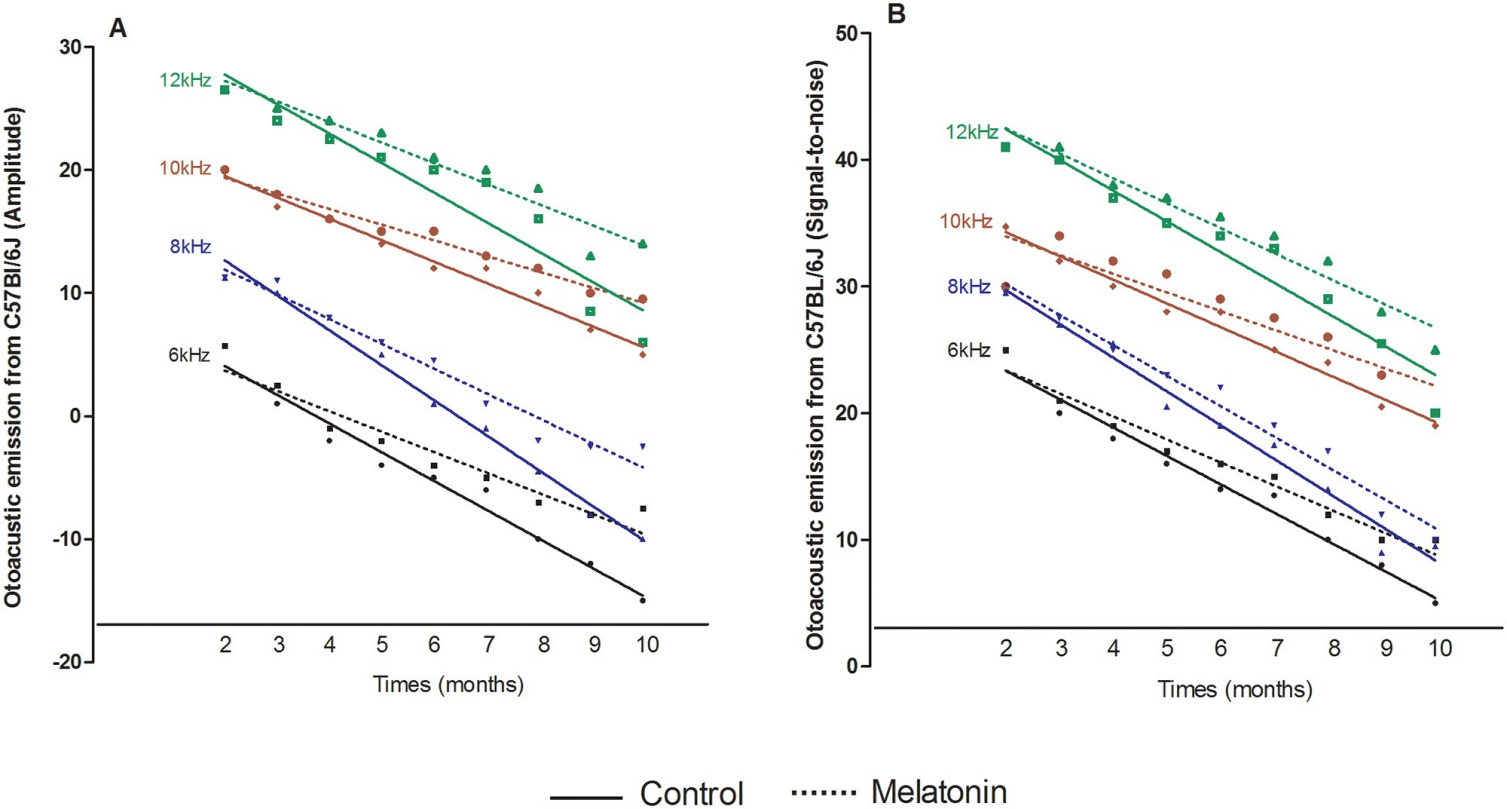
Linear regression adjusted lines. Adjusted lines of the variation of (A) amplitude of the otoacoustic emission and (B) signal-to-noise ratio obtained monthly in the control group (continuous line) and melatonin-treated (dotted line) group. Frequencies of 6 (black), 8 (blue), 10 (red) and 12 kHz (green) are shown.

The data showed that both groups (CG and MG) had decreased DPOAE levels during the study period, suggesting a reduction in the number of cochlear hair cells (Fig 1A and 1B). The linear regression suggests that the amplitude and the signal-to-noise ratio decreased over time for both groups (p<0.001; r^2^>0.93).

**Fig 2:**
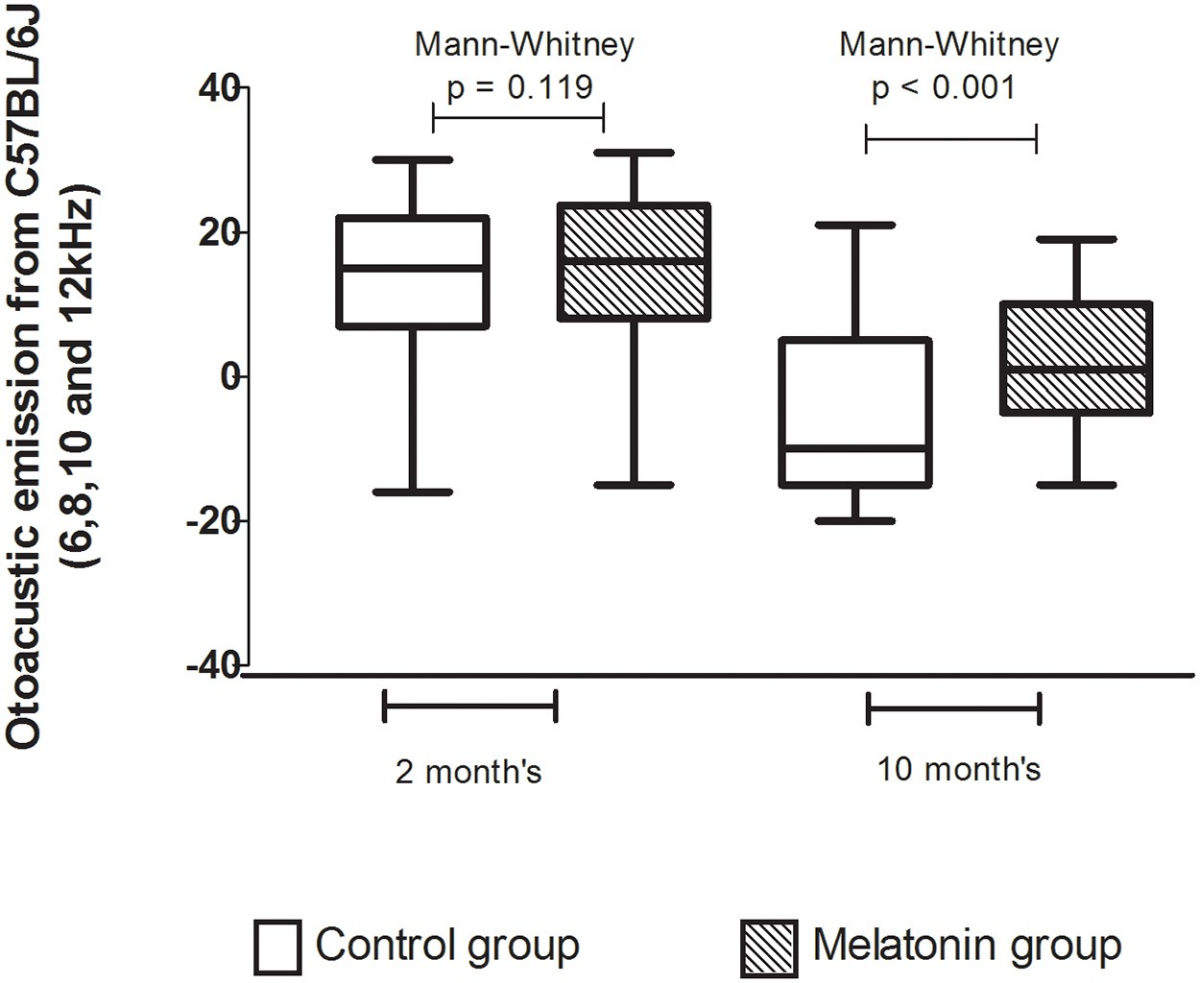
Boxplot of medians, quartiles, maximum and minimum values of the 6, 8 10 and 12 kHz frequency ranges of the control and melatonin-treated groups.

Fig 2 shows that there was a reduction in the otoacoustic emissions over time in both groups when compared to the tests performed during the 2^nd^ month of life. In the 10^th^ month, differences were noticeable between the two groups. Notably, the CG presented lower DPOAE values when compared to the MG (one-tailed Mann-Whitney, p<0.001).

## Discussion

The use of melatonin to delay age-related deafness may be a novel, alternative treatment for clinical practice. In this study, daily administration of 10 mg/kg of melatonin in ARHL-susceptible animals delayed this condition, and mice had improved DPOAE values at all tested frequencies compared to the control group.

The C57BL/6J mice used in this study exhibit an ARHL pattern due to a mutation in the *Cdh23* gene, which encodes cadherin 23 (otocaderine). This mutation involves the replacement of a guanine (G) by an adenine (A) in the 7^th^ exon. Cadherin 23 is an adhesion glycoprotein expressed between cells in the inner ear sensory epithelium, and is necessary for the formation and functionality of stereocilia bundles [26,27]. A study done in humans shows that the single nucleotide G>A polymorphism is associated with ARHL [28]. Reduction or lack of otocaderine predisposes cell stereocilia to cell damage, causing hair cell apoptosis over time. In this animal model, it is possible to capture the DPOAE around the 21st–28th day of life. Throughout their lifespan and with the gradual decline of cochlear activity, it is possible to observe the reduction of these emissions [24].

DPOAE levels were shown to be significantly altered at 12 months in this strain, and at 20 months, it was no longer possible to capture them [29]. In the present study, it was possible to follow the gradual decline in DPOAE levels over the lifespan of the mice in both groups, similar to humans during the ARHL process. Interestingly, there was a sharp decrease during the 10th month in both the response amplitude analysis and in the signal-to-noise ratio; however, the melatonin group had better responses than the control group at all evaluated frequencies.

In this study, the difference between the groups could be verified initially at higher frequencies (10 and 12 kHz) from the 6th month onwards, when the otoprotective effect of the melatonin was perceived. At frequencies of 6 and 8 kHz, this effect was perceived at the 10th month, both in amplitude and signal-to-noise ratio amplitudes.

Several studies [30,31] have indicated that ARHL starts at higher frequencies, consistent with our results in both groups. This fact can be explained by the occurrence of cell loss at the cochlear basal turn, where there is an intense contact between the sound and the cochlea, so these cells are more exposed to damage as well as to oxidative stress and the cell aging process.

Aging is a process of oxidative damage that occurs due to the accumulation of reactive oxygen species in various body tissues, including the cochlea. Cellular damage that occurs due to reactive oxygen species accumulation causes mitochondrial dysfunction with reduced energy production and consequent tissue dysfunction [32]. Several studies have shown that melatonin levels in the body significantly decrease with age [33,34]. The results obtained in this study indicate that clinical use of this antioxidant may be an alternative therapeutic to delay the effects of ARHL in humans. Considering its ability to act as an electron donor in non-enzymatic processes, it has an important role in neutralizing free radicals and stimulating the activity of various antioxidant enzymes.

Oxidative damage to DNA increases with age and may have a causal relationship with ARHL through the induction of apoptosis [22,35]. A study on the use of dietary supplementation with various antioxidants has shown that mitochondrial antioxidants reduced cochlear cell death and delayed the onset of ARHL [22].

In another study, dogs were fed a combination of antioxidants in the last 3 years of their lives. Interestingly, these animals had lower levels of degeneration in the spiral ganglion cells and vascular stria when compared to control animals [36]. Our study found that the changes caused by ARHL were reduced due to the continuous treatment of melatonin at a 10 mg/kg/day dose over 10 months of life, since we observed that the animals in the MG had better “aging values” of amplitude and signal-to-noise ratio when compared to the CG animals.

The effects of antioxidants (melatonin, vitamin C, E and lazaroids) and caloric restriction on the development of presbycusis were previously studied using a 0.1 mg/day melatonin dose offered through an subcutaneous implant in the animal. The author concluded that the group that developed the lowest rates of presbycusis was that under caloric restriction. The melatonin-treated group showed a statistical improvement over the control group at all auditory frequencies except 12 kHz (3, 6, 9, 12 and 18 kHz were tested). All groups provided antioxidants had better hearing sensitivity compared to the control group [37]. In this study, the frequencies analyzed were 6, 8 10 and 12 kHz, and in all of them, the MG showed better results than the CG.

An important aspect of this study was the dose and the form of administration; 10 mg/kg/day of melatonin was delivered orally, in an attempt to replicate how the substance would be used clinically by humans. In several studies, antioxidant administration was performed through the diet, using subcutaneous implantation, or mixed in the drinking water [22,36,37]. The option of administering the antioxidant directly into the animal’s mouth ensured the ingestion of the exact volume offered daily over time.

A limitation of this study is that no histological analysis was performed, which would allow the comparison of clinical exam data with objective images. In the future, we intend to perform this analysis to better understand the intracellular and tissue effects of melatonin on the cochlear cells.

Few studies have been performed using melatonin as a protective factor in ARHL. However, as a safe substance that has not shown adverse effects on humans or animals, its use may contribute to improving the quality of life of the elderly population and allow a level of independence in performing activities of daily living.

## Conclusion

Mice treated with oral melatonin at a dose of 10 mg/kg/day maintained higher DPOAE amplitude and signal-to-noise ratio at the end of 10 months of life compared to a control group. Animals in both groups had a reduction in these parameters throughout their lives. It cannot be stated that continuous use of melatonin prevents the onset of ARHL; however, it can be suggested that the use of this substance delays the onset of the presbycusis.

## Supporting information

S1 File Outcomes of the study. (XLSX)

## References

[1] de Mello JM, Della-Rosa VA, Carvallo RMM. Distortion-product otoacoustic emissions at ultra-high frequencies in parents of individuals with autosomal recessive hearing loss. CoDAS. 2014;26(1):3–9. Available from: http://www.scielo.br/scielo.php?script=sci_arttext&pid=S2317-17822014000100003&lng=en&tlng=en 24714853

[2] Boechat EM. Tratado de Audiologia. 2 ed. Koogan G, editor. Rio de Jnaeiro; 2015. CAP.37.

[3] Yamasoba T, Someya S, Yamada C, Weindruch R, Prolla TA, Tanokura M. Role of mitochondrial dysfunction and mitochondrial DNA mutations in age-related hearing loss. Hear Res. 2007;226(1–2):185–193. 10.1016/j.heares.2006.06.004 16870370

[4] Tretbar K, Basilowski M, Wiedmann K, Bartels C, Heßmann P, Kownatka M, et al Lebensqualität und Depression bei Hörminderung. HNO. 2019;67(1):36–44. Available from: http://link.springer.com/10.1007/s00106-018-0576-4 30324556 10.1007/s00106-018-0576-4

[5] Gorecka MM, Vasylenko O, Espenes J, Waterloo K, Rodríguez-Aranda C. The impact of age-related hearing loss and lateralized auditory attention on spatiotemporal parameters of gait during dual-tasking among community dwelling older adults. Exp Gerontol. 2018;111:253–262. Available from: https://linkinghub.elsevier.com/retrieve/pii/S0531556518300743 30056101 10.1016/j.exger.2018.07.015

[6] Dong Y, Guo C, Chen D, Chen S, Peng Y, Song H, et al Association between age-related hearing loss and cognitive decline in C57BL/6J mice. Mol Med Rep. 2018; Available from: http://www.spandidos-publications.com/10.3892/mmr.2018.9118 10.3892/mmr.2018.9118 29901198

[7] Tavanai E, Mohammadkhani G. Role of antioxidants in prevention of age-related hearing loss: a review of literature. Eur Arch Oto-Rhino-Laryngology. 2017;274(4):1821–1834. 10.1007/s00405-016-4378-6 27858145

[8] Bernabei R, Bonuccelli U, Maggi S, Marengoni A, Martini A, Memo M, et al Hearing loss and cognitive decline in older adults: questions and answers. Aging Clin Exp Res. 2014;26(6):567–573. Available from: http://link.springer.com/10.1007/s40520-014-0266-3 25281432 10.1007/s40520-014-0266-3

[9] Morrill S, He DZZ. Apoptosis in inner ear sensory hair cells. J Otol. 2017;12(4):151–164. Available from: https://linkinghub.elsevier.com/retrieve/pii/S1672293017300752 29937851 10.1016/j.joto.2017.08.001 PMC6002637

[10] Grivicich I, Regner A, da Rocha AB. Morte Celular por Apoptose. Rev Bras Cancrol. 2007;53(3):335–343. Available from: http://www1.inca.gov.br/rbc/n_53/v03/pdf/revisao4.pdf

[11] Wong ACY, Ryan AF. Mechanisms of sensorineural cell damage, death and survival in the cochlea. Front Aging Neurosci. 2015;7(APR):1–15. 25954196 10.3389/fnagi.2015.00058 PMC4404918

[12] Lasisi TJ, Lasisi AO. Evaluation of serum antioxidants in age-related hearing loss. Aging Clin Exp Res. 2015;27(3):265–269. Available from: http://link.springer.com/10.1007/s40520-014-0282-3 25362621 10.1007/s40520-014-0282-3

[13] Ackah SEH, Juhn SK, Huang TC, Wiedmann TS. A combination antioxidant therapy prevents age-related hearing loss in C57BL/6 mice. Otolaryngol Neck Surg. 2010;143(3):429–434. Available from: http://journals.sagepub.com/doi/10.1016/j.otohns.2010.04.266 10.1016/j.otohns.2010.04.266 20723783

[14] Kamogashira T, Fujimoto C, Yamasoba T. Reactive Oxygen Species, Apoptosis, and Mitochondrial Dysfunction in Hearing Loss. Biomed Res Int. 2015;2015:1–7. Available from: http://www.hindawi.com/journals/bmri/2015/617207/10.1155/2015/617207 PMC4385658 25874222

[15] Volquind D. Daniel Volquind Efeitos da melatonina sobre a inflamação e o estresse. 2016;

[16] Sletten TL, Vincenzi S, Redman JR, Lockley SW, Rajaratnam SMW. Timing of Sleep and Its Relationship with the Endogenous Melatonin Rhythm. Front Neurol. 2010;1(November):1–8. Available from: http://journal.frontiersin.org/article/10.3389/fneur.2010.00137/abstract 21188265 10.3389/fneur.2010.00137 PMC3008942

[17] Reiter RJ, Tan DX, Rosales-Corral S, Galano A, Zhou XJ, Xu B. Mitochondria: Central organelles for melatonins antioxidant and anti-Aging actions. Molecules. 2018;23(2):1–25. 10.3390/molecules23020509 PMC6017324 29495303

[18] Rasmussen DD, Boldt BM, Wilkinson C, Yellon SM, Matsumoto AM. Daily Melatonin Administration at Middle Age Suppresses Male Rate Visceral Fat, Plasma Leptin, and Plasma Insulin to Youthful Levels. Endocrinology. 1999;140(2):1009–1012. Available from: https://academic.oup.com/endo/article-lookup/doi/10.1210/endo.140.2.6674 9927336 10.1210/endo.140.2.6674

[19] Wolden-Hanson T, Mitton DR, McCants RL, Yellon SM, Wilkinson CW, Matsumoto AM, et al Daily Melatonin Administration to Middle-Aged Male Rats Suppresses Body Weight, Intraabdominal Adiposity, and Plasma Leptin and Insulin Independent of Food Intake and Total Body Fat1. Endocrinology. 2000;141(2):487–497. Available from: https://academic.oup.com/endo/article/141/2/487/2987744 10650927 10.1210/endo.141.2.7311

[20] Karlidağ T, Yalçin Ş, Öztürk A, Üstündağ B, Gök Ü, Kaygusuz İ, et al The role of free oxygen radicals in noise induced hearing loss: effects of melatonin and methylprednisolone. Auris Nasus Larynx. 2002;29(2):147–152. Available from: https://linkinghub.elsevier.com/retrieve/pii/S0385814601001377 11893449 10.1016/s0385-8146(01)00137-7

[21] De Araujo JG, Serra LSM, Lauand L, Kückelhaus SAS, Sampaio ALL. Protective Effect of Melatonin on Cisplatin-induced Ototoxicity in Rats. Anticancer Res. 2019;39(5):2453–2458. Available from: http://ar.iiarjournals.org/lookup/doi/10.21873/anticanres.13364 31092439 10.21873/anticanres.13364

[22] Someya S, Xu J, Kondo K, Ding D, Salvi RJ, Yamasoba T, et al Age-related hearing loss in C57BL/6J mice is mediated by Bak-dependent mitochondrial apoptosis. Proc Natl Acad Sci. 2009;106(46):19432–19437. Available from: http://www.pnas.org/cgi/doi/10.1073/pnas.0908786106 19901338 10.1073/pnas.0908786106 PMC2780799

[23] Laboratory J. No Title [Internet]. [cited 2018 Jan 3]. p. jax.org. jax.org

[24] Narui Y, Minekawa A, Iizuka T, Furukawa M, Kusunoki T, Koike T, et al Development of distortion product otoacoustic emissions in C57BL/6J mice. Int J Audiol. 2009;48(8):576–581. Available from: http://www.tandfonline.com/doi/full/10.1080/14992020902858959 19842812 10.1080/14992020902858959

[25] Sociedade Brasileira de Ciência em Animais de Laboratório—COBEA.

[26] Liu S, Li S, Zhu H, Cheng S, Zheng QY. A mutation in the cdh23 gene causes age-related hearing loss in Cdh23nmf308/nmf308 mice. Gene. 2012;499(2):309–317. Available from: https://linkinghub.elsevier.com/retrieve/pii/S0378111912001400 22326520 10.1016/j.gene.2012.01.084 PMC3526976

[27] Mock BE, Vijayakumar S, Pierce J, Jones TA, Jones SM. Differential effects of Cdh23 753A on auditory and vestibular functional aging in C57BL/6J mice. Neurobiol Aging. 2016;43:13–22. Available from: https://linkinghub.elsevier.com/retrieve/pii/S0197458016002116 27255811 10.1016/j.neurobiolaging.2016.03.013 PMC4893173

[28] Bowl MR, Dawson SJ. The Mouse as a Model for Age-Related Hearing Loss—A Mini-Review. Gerontology. 2014;61(2):149–157. Available from: https://www.karger.com/Article/FullText/368399 25471225 10.1159/000368399

[29] Parham K. Distortion product otoacoustic emissions in the C57BL / 6J mouse model of age-related hearing loss. Hear Res. 1997;112(June):216–234. 9367243 10.1016/s0378-5955(97)00124-x

[30] Bowl MR, Dawson SJ. Age-Related Hearing Loss. Cold Spring Harb Perspect Med. 2019;9(8):a033217 Available from: http://perspectivesinmedicine.cshlp.org/lookup/doi/10.1101/cshperspect.a033217 30291149 10.1101/cshperspect.a033217 PMC6671929

[31] Polanski JF, Cruz OL. Evaluation of antioxidant treatment in presbyacusis: Prospective, placebo-controlled, double-blind, randomised trial. J Laryngol Otol. 2013;127(2):134–141. 10.1017/S0022215112003118 23318104

[32] Fujimoto C, Yamasoba T. Oxidative Stresses and Mitochondrial Dysfunction in Age-Related Hearing Loss Oxid Med Cell Longev. 2014;2014:1–6. Available from: http://www.hindawi.com/journals/omcl/2014/582849/10.1155/2014/582849 PMC4106174 25110550

[33] García JJ, López-Pingarrón L, Almeida-Souza P, Tres A, Escudero P, García-Gil FA, et al Protective effects of melatonin in reducing oxidative stress and in preserving the fluidity of biological membranes: a review. J Pineal Res. 2014;56(3):225–237. Available from: http://doi.wiley.com/10.1111/jpi.12128 24571249 10.1111/jpi.12128

[34] Cipolla-Neto J, do Amaral FG. Melatonin as a Hormone: New Physiological and Clinical Insights. Endocr Rev. 2018;39(6):990–1028. Available from: https://academic.oup.com/edrv/article/39/6/990/5094958 30215696 10.1210/er.2018-00084

[35] Park S-N, Back S-A, Park K-H, Kim D-K, Park SY, Oh J-H, et al Comparison of Cochlear Morphology and Apoptosis in Mouse Models of Presbycusis. Clin Exp Otorhinolaryngol. 2010;3(3):126 Available from: http://e-ceo.org/journal/view.php?doi=10.3342/ceo.2010.3.3.126 20978629 10.3342/ceo.2010.3.3.126 PMC2958502

[36] Le T, Keithley EM. Effects of antioxidants on the aging inner ear. Hear Res. 2007;226(1–2):194–202. 10.1016/j.heares.2006.04.003 16843623

[37] Seidman MD. Effects of dietary restriction and antioxidants on presbyacusis. Laryngoscope. 2000;110(May):727–738. 10807352 10.1097/00005537-200005000-00003

